# Construction of a Prognostic Gene Signature Associated with Immune Infiltration in Glioma: A Comprehensive Analysis Based on the CGGA

**DOI:** 10.1155/2021/6620159

**Published:** 2021-03-13

**Authors:** Xiaoxiang Gong, Lingjuan Liu, Jie Xiong, Xingfang Li, Jie Xu, Yangyang Xiao, Jian Li, Xuemei Luo, Dingan Mao, Liqun Liu

**Affiliations:** ^1^Department of Pediatrics, The Second Xiangya Hospital, Central South University, Changsha 410011, Hunan, China; ^2^Childern's Brain Development and Brain Injury Research Office, The Second Xiangya Hospital, Central South University, Changsha 410011, Hunan, China

## Abstract

**Background:**

Tumor microenvironment (TME) is closely related to the progression of glioma and the therapeutic effect of drugs on this cancer. The aim of this study was to develop a signature associated with the tumor immune microenvironment using machine learning.

**Methods:**

We downloaded the transcriptomic and clinical data of glioma patients from the Chinese Glioma Genome Atlas (CGGA) databases (mRNAseq_693). The single-sample Gene Set Enrichment Analysis (ssGSEA) database was used to quantify the relative abundance of immune cells. We divided patients into two different infiltration groups via unsupervised clustering analysis of immune cells and then selected differentially expressed genes (DEGs) between the two groups. Survival-related genes were determined using Cox regression analysis. We next randomly divided patients into a training set and a testing set at a ratio of 7 : 3. By integrating the DEGs into least absolute shrinkage and selection operator (LASSO) regression analysis in the training set, we were able to construct a 15-gene signature, which was validated in the testing and total sets. We further validated the signature in the mRNAseq_325 dataset of CGGA.

**Results:**

We identified 74 DEGs associated with tumor immune infiltration, 70 of which were significantly associated with overall survival (OS). An immune-related gene signature was established, consisting of 15 key genes: adenosine triphosphate (ATP)-binding cassette subfamily C member 3 (*ABCC3*), collagen type IV alpha 1 chain (*COL4A1*), podoplanin (*PDPN*), annexin A1 (*ANXA1*), *COL4A2*, insulin-like growth factor binding protein 2 (*IGFBP2*), serpin family A member 3 (*SERPINA3*), CXXC-type zinc finger protein 11 (*CXXC11*), junctophilin 3 (*JPH3*), secretogranin III (*SCG3*), secreted protein acidic and rich in cysteine (SPARC)-related modular calcium-binding protein 1 (*SMOC1*), Cluster of Differentiation 14 (*CD14*), *COL1A1*, S100 calcium-binding protein A4 (*S100A4*), and transforming growth factor beta 1 (*TGF-β1*). The OS of patients in the high-risk group was worse than that of patients in the low-risk group. GSEA showed that interleukin-6 (*IL-6*)/Janus kinase (*JAK*)/signal transducer and activator of transcription (*STAT3*) signaling, interferon gamma (IFN-*γ*) response, angiogenesis, and coagulation were more highly enriched in the high-risk group and that oxidative phosphorylation was more highly enriched in the low-risk group.

**Conclusion:**

We constructed a stable gene signature associated with immune infiltration to predict the survival rates of glioma patients.

## 1. Introduction

Glioma is the most common type of primary brain tumor. As it is highly infiltrative and aggressive, survival time is short, even after combined treatment with surgery, radiotherapy, and chemotherapy. The World Health Organization (WHO) classification system divides glioma into low-grade (I-II) and high-grade (III-IV) based on the absence or presence of anaplastic features and histological characteristics with molecular features, including isocitrate dehydrogenase 1 (IDH1) mutation and 1p/19q codeletion [1, 2]. Despite all that, prognostic prediction and improvement of treatment efficiency in glioma remain challenging. Therefore, identifying novel prognostic biomarkers and therapeutic targets is imperative in this cancer.

Recent research has shown that immune infiltration plays a crucial role in cancer tumorigenesis and progression [3]. Tumor immune infiltration involves multiple immune cells whose functions are significantly altered in glioma. The research of Tian et al. notes that M0 and M2 tumor-associated macrophages (TAMs) play a protumor role, while M1 TAMs play an antitumor role [4]. The glioblastoma microenvironment has been demonstrated to increase counts of myeloid-derived suppressor cells, which block T cell and natural-killer (NK) cell functions, resulting in an immunocompromised microenvironment [5]. By secreting cytokines such as transforming growth factor beta (TGF-*β*), the glioma microenvironment favors recruitment and survival of regulatory T cells (Tregs), which are associated with worse prognosis in many cancers [6–8]. Therefore, targeting these cells in treatment is expected to shift the glioma tumor microenvironment (TME) from a “cold” to a “hot” phenotype [9].

With the rapid development of transcriptome sequencing, analytical methods or databases such as CIBERSORT, single-sample Gene Set Enrichment Analysis (ssGSEA), ESTIMATE algorithm, and Tumor Immune Estimation Resource (TIMER) have been developed to evaluate tumor immune infiltration [10–13]. Signatures based on immune infiltration also show promising predictive value in tumor prognosis and immunotherapeutic response. Most studies on immune-related prognostic biomarkers in glioma have focused on tumor-infiltrating cells. However, the definition of immune cell type depends on protein molecular-weight markers; immune-related biomarkers rather than immune cells constitute another way to view the TME. Tian et al. constructed a classifier of immune-relevant long noncoding ribonucleic acids (lncRNAs) using immune gene sets from the Molecular Signatures Database (MSigDB) to predict glioma prognosis [14]. Based on the ESTIMATE algorithm, Zhang et al. constructed a four-gene signature involved in immune infiltrssation in glioblastoma [15]. Xiao et al. provided an immune-related gene model for risk stratification in lower-grade glioma using CIBERSORT and TIMER [16]. To date, no signature associated with immune infiltration in glioma based on ssGSEA has been explored.

In this study, we conducted a comprehensive analysis of the immune infiltration landscape based on a dataset from the Chinese Glioma Genome Atlas (CGGA) databases and established a predictive model. Our results demonstrated that the risk score of the signature was an independent prognostic factor for glioma patients.

## 2. Methods

### 2.1. Data Acquisition

We drew the transcriptomic and clinical data of glioma patients from the public CGGA (http://www.cgga.org.cn/) databases mRNAseq_693 and mRNAseq_325 [17, 18]. Patients with complete overall survival (OS) information and gene expression profiles were included in our study. We used fragments per kilobases per million reads (FPKM) to estimate RNA expression.

### 2.2. Abundance of Immune Cells

In this study, we applied ssGSEA to quantify the relative abundance of 28 immune cells based on gene sets obtained from the TISIDB database, an integrated repository portal for tumor immune system interactions [12]. We evaluated 28 immune cell types, including monocytes, central-memory Cluster of Differentiation 4 (CD4) T cells, plasmacytoid dendritic cells, immature dendritic cells, activated dendritic cells, CD56^dim^ natural-killer (NK) cells, CD56^bright^ NK cells, *γδ* T cells, NK cells, myeloid-derived suppressor cells, central-memory CD8 T cells, effector memory CD4 T cells, T follicular helper (T_fh_) cells, effector memory CD8 T cells, macrophage, activated CD8 T cells, NK T cells, type 1T helper (T_h_1) cells, regulatory T cells (Tregs), T_h_2 cells, memory B cells, immature B cells, T_h_17 cells, mast cells, activated CD4 T cells, eosinophils, activated B cells, and neutrophils. Unsupervised classification of the glioma cohort was conducted through *k*-means clustering on ssGSEA enrichment score [19, 20]. We used the R software (https://www.r-project.org) package ConsensusClusterPlus to implement an unsupervised consensus approach and divided glioma patients into two immune-infiltrating subtypes according to infiltrating densities of immune cells [21].

### 2.3. Differentially Expressed lncRNAs and mRNAs

To identify lncRNAs and messenger RNAs (mRNAs) associated with immune infiltration, we compared gene expression between different samples using the ballgown method [22]. An adjusted *P* value of <0.05 and log2 fold change (|log2FC|) > 1 were set as the cutoff criteria for differentially expressed lncRNAs, while *P* < 0.05 and |log2FC| > 2 were set as the cutoff criteria for differentially expressed mRNAs. Unsupervised classification of the glioma cohort was conducted via *k*-means clustering on the expression of differentially expressed mRNA.

### 2.4. GO and KEGG Pathway Analysis

To analyze the possible functions of these differentially expressed mRNAs, we used the R package clusterProfiler to conduct Gene Ontology (GO) and Kyoto Encyclopedia of Genes and Genomes (KEGG) enrichment analyses [23, 24]. GO enrichment was carried out from the following three aspects: biological processes (BP), cellular components (CC), and molecular functions (MF). KEGG enrichment was mainly focused on molecular mechanisms and metabolic pathways. The top 15 items of GO categories and KEGG pathways are shown in the bubble maps.

### 2.5. Univariate Cox and LASSO Regression Analyses

To obtain immune- and survival-related genes, we analyzed differentially expressed mRNAs via univariate Cox regression using the R package survival. Candidate mRNAs were selected if *P* < 0.01. Using R software, we randomly divided CGGA glioma patients into a training set and a testing set at a ratio of 7 : 3. The abovementioned immune- and survival-related genes were integrated into least absolute shrinkage and selection operator (LASSO) regression analysis in the training set as calculated by the R package glmnet [25]. The signature to calculate the risk score of each patient equaled the sum of the respective LASSO coefficients multiplied by mRNA expression level. We then validated the signature in the testing and total sets. Next, all patients were split into low-risk and high-risk groups according to median risk score.

### 2.6. GSEA

Based on hallmark gene sets, we used GSEA to identify differentially regulated pathways between the low-risk and high-risk groups [26, 27]. Enriched pathways with a normal *P* value of <0.05 and false discovery rate (FDR) *Q*-value of <0.25 were considered statistically significant.

### 2.7. Statistical Analyses

For comparisons between two groups, we used Student's *t*-test and Wilcoxon test. For comparisons among three groups, we used one-way ANOVA and Kruskal–Wallis test. Spearman's correlation coefficient (SCC) was used to calculate coefficients between two parameters and Fisher's exact test to estimate correlations between mRNAs and the terms of GO and KEGG analyses. We used the univariate Cox model to determine independent prognostic factors. Receiver operating characteristic (ROC) analysis and area under the curve (AUC) were used to evaluate the specificity and sensitivity of the signature. The survival difference between the two groups was demonstrated by Kaplan–Meier (KM) curves.

## 3. Results

### 3.1. The Landscape of Tumor-Infiltrating Immune Cells in Glioma

**Figure 1:**
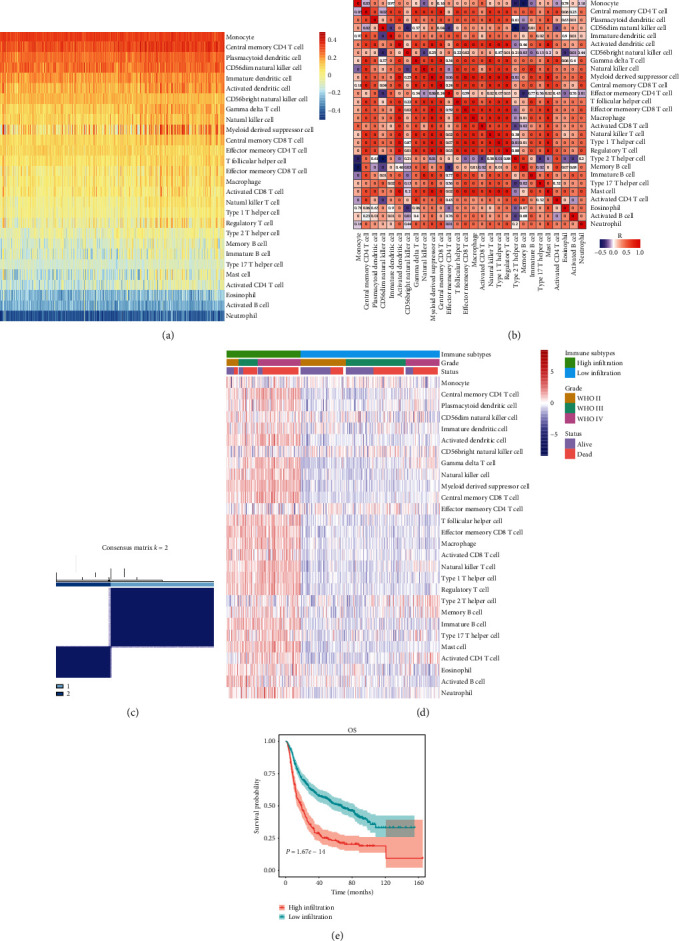
(a) Enrichment level of infiltrating immune cells in glioma based on ssGSEA data. (b) Correlation matrix of 28 immune cells. (c) Consensus clustering matrix for *k* = 2, which was the optimal cluster number in the cohort. (d) Unsupervised clustering of 28 immune cells. Clinical data, including tumor grade and survival status, are also shown. (e) Survival analysis of the two groups of patients divided by immune infiltration levels.

We analyzed enrichment levels of 28 immune cells in glioma using immune-associated gene sets in ssGSEA. The comprehensive immune landscape of the glioma cohort is depicted in the form of a heatmap (Figure 1(a)). Results revealed that densities of monocytes, CD56^dim^ NK cells, plasmacytoid dendritic cells, immature dendritic cells, and central-memory CD4 T cells were high. In contrast, densities of eosinophils, neutrophils, and activated B cells were low.  Figure 1(b) indicates the correlation coefficient among the 28 immune cell types. We can find some highly correlated immune cell couples in this figure, such as T_h_2 cells/CD56 NK cells, monocyte/memory B cells and Tregs/activated dendritic cells. We clustered glioma samples according to the ssGSEA scores of these immune-associated gene sets and divided them into two distinct clusters: high_infiltration and low_infiltration (Figures 1(c)–1(d)). Results confirmed that patients with high-infiltration TMEs had worse clinical outcomes than those with low-infiltration TMEs (Figure 1(e)). These data also proved the importance of subgrouping as a basis for immunogenomic profiling.

### 3.2. Identification of Differentially Expressed lncRNAs

**Figure 2:**
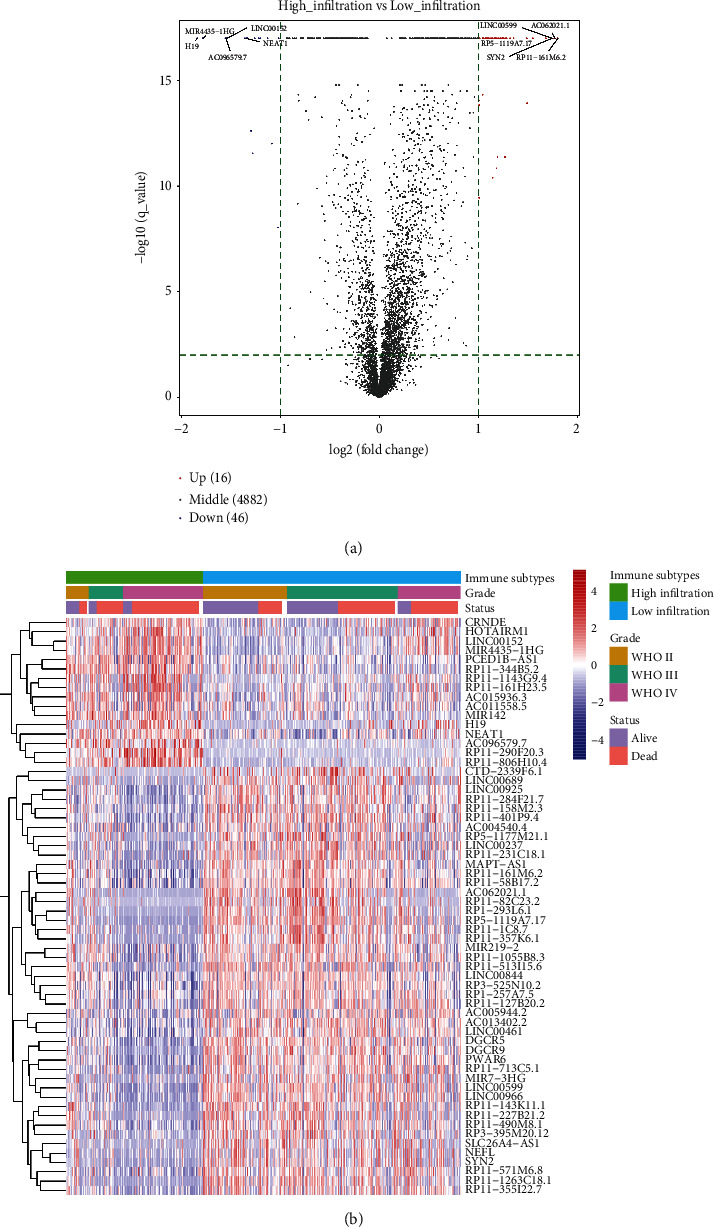
(a) Volcano plot of differently expressed lncRNAs. Spots on the right represent upregulated lncRNAs in the high-infiltration group; spots on the left represent upregulated lncRNAs in the low-infiltration group. (b) Heatmap of differently expressed lncRNAs. Clinical data, including tumor grade and survival status, are also shown.

Because OS differed remarkably between the low-infiltration and high-infiltration groups, we speculated that transcriptomic differences between the two groups might also exist. Previous studies demonstrated that both lncRNAs and microRNAs (miRNAs) play essential roles in tumor immune responses [28–31]. In this study, we explored the transcriptome of the CGGA cohort and compared differentially expressed lncRNAs between the two infiltration-level groups. In the high-infiltration group, 16 immune-related lncRNAs were upregulated, while in the low-infiltration group, 46 were upregulated (Table 1; Figure 2). *RP11-161M6.2*, *AC62021.1*, synaptophysin 2 (*SYN2*), long intergenic noncoding RNA 152 (*LINC00599*), and *RP5-1119A7.17* were the top five most highly expressed lncRNAs in the low-infiltration group, while *H19,* microRNA 4435-1 host gene (*MIR4435-1HG*), *AC096579.7*, *LINC00152,* and nuclear paraspeckle assembly transcript 1 (*NEAT1*) were the top five in the high-infiltration group.

### 3.3. Identification of Differentially Expressed mRNAs

**Figure 3:**
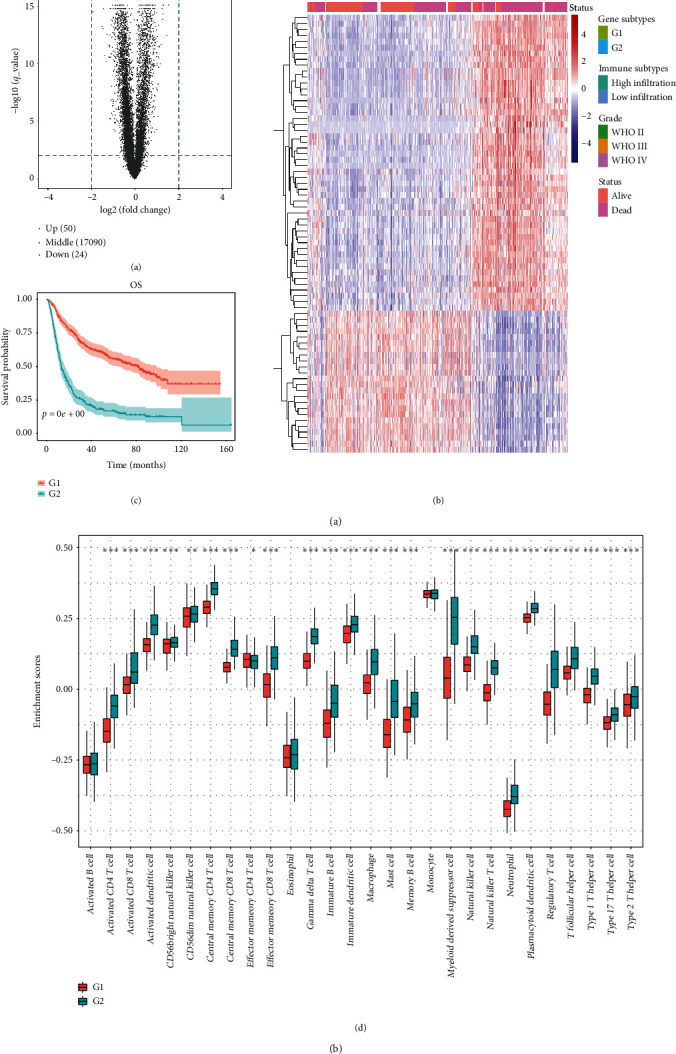
(a) Volcano plot of differentially expressed mRNAs. Spots on the right represent upregulated genes in the low-infiltration group; spots on the left represent upregulated genes in the high-infiltration group. (b) Unsupervised clustering of 74 DEGs. Clinical data, including tumor grade and survival status, are also shown. (c) Survival analysis of the two gene subtypes. (d) Comparison of immune cell abundances between the two gene subtypes ( ^*∗*^*P* < 0.05,  ^*∗∗∗*^*P* < 0.001).

By comparing differentially expressed mRNAs between the low- and high-infiltration groups, we obtained 50 immune-related mRNAs that were upregulated in the high-infiltration group and 24 that were upregulated in the low-infiltration group (Table 2; Figure 3(a)). Neuron-specific gene 2 (*NSG2*), glutamate ionotropic receptor N-methyl-D-aspartate type subunit 1 (*GRIN1*), complexin-2 (*CPLX2*), chromogranin A (*CHGA*), and synaptosomal-associated protein, 25 kDa (*SNAP25*), were the top five in the low-infiltration group, while chitinase-3-like proteins 1 and 2 (*CHI3L1*, *CHI3L2*), Spen paralog and ortholog C-terminal domain containing 1 (*SPOCD1*), lactoferrin (*LTF*), and serpin family A member 3 (*SERPINA3*) were the top five in the high-infiltration group. Next, through unsupervised clustering of differentially expressed mRNAs, we classified patients into two gene subgroups: G1 and G2 (Figure 3(b)). The survival probability of the G1 subgroup was remarkably higher than that of the G2 subgroup (Figure 3). After comparing the relative quantity of the 28 immune cells between the two subgroups, we found that the infiltration level of most immune cells was apparently higher in the G2 than the G1 subgroup (Figure 3).

### 3.4. Construction of an Immune Infiltration-Related Gene Signature

**Figure 4:**
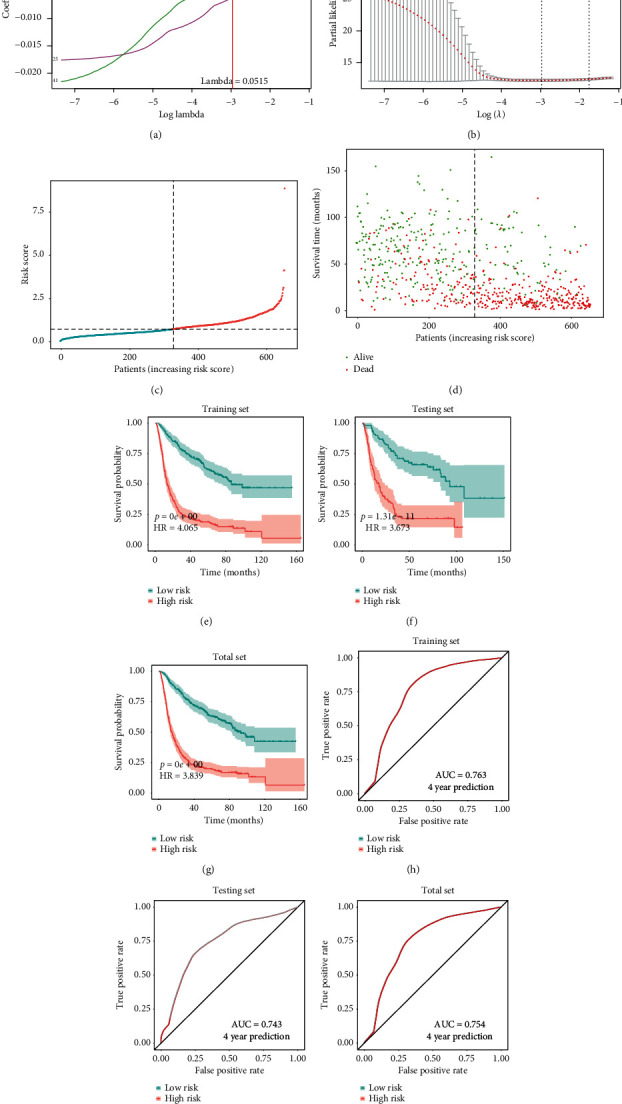
(a) LASSO coefficient profiles of the 70 survival-associated genes. (b) Selection of optimal *λ* value in the LASSO model for glioma. (c) Distribution of risk score in the cohort. (d) Scatter plot of patients with different survival statuses. (e–g) KM survival analyses of the survival difference between the low- and high-risk groups in the training, testing, and total sets. (h–j) ROC curves show the prediction efficiency of the signature in the training, testing, and total sets.

We conducted univariate Cox regression analysis of the 74 putative immune-related genes. A total of 70 genes significantly associated with survival were identified; they are listed in Table 3 (*P* < 0.01). We randomly divided glioma patients into a training set and a testing set at a ratio of 7 : 3. Next, we integrated the 70 genes into the LASSO Cox regression algorithm. A well balanced prognostic model was obtained after 1000 iterations, and we selected 15 immune-related genes to construct the signature (Figures 4(a)–4(d)). The full names of the genes and their LASSO coefficients are listed in Table 4, and the survival curves of the genes are displayed in Figure S1. Next, we calculated risk score for each glioma patient and categorized all patients into low-risk and high-risk groups based on median risk score. KM curve analysis demonstrated that patients in the high-risk group had significantly worse OS than those in the low-risk group (Figure 4(e)). After application of the ROC curve, the AUC for 4-year prediction was 0.763 in the training set, indicating excellent prediction efficiency (Figure 4(h)). Then, to validate this signature, we also calculated the risk scores of patients in the testing and total sets. Survival time was remarkably different between the low-risk and high-risk groups, both in the testing set and the total set (Figures 4(f)–4(g)). The AUC for 4-year prediction was 0.743 in the testing set and 0.754 in the total set (Figures 4(i)–4(j)).

### 3.5. Application of the Immune Signature in Glioma Subgroup Analysis

**Figure 5:**
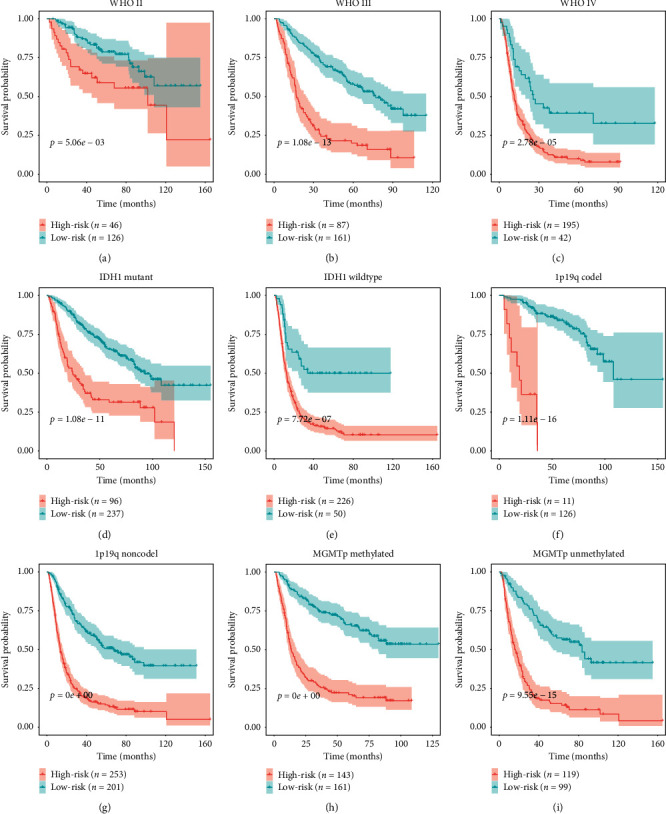
Confirmation of the signature via patient stratification based on specific pathological and molecular features. (a) Grade II; (b) grade III; (c) grade IV; (d) mutated *IDH1*; (e) wild-type *IDH1*; (f) 1p/19q codeletion; (g) 1p/19q noncodeletion; (h) MGMT promoter methylated status; (i) MGMT promoter unmethylated status.

**Figure 6:**
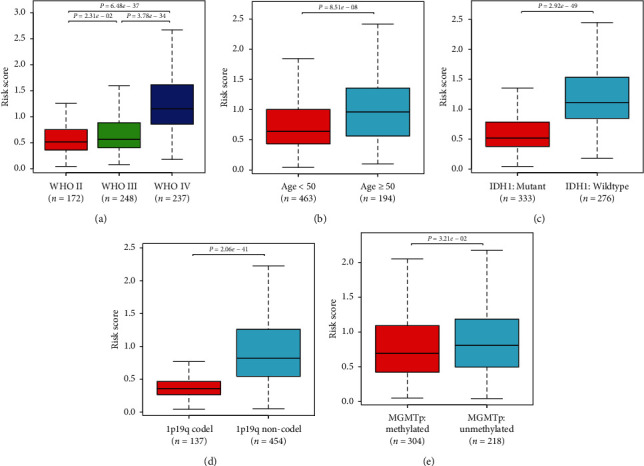
Correlations between risk score and clinicopathological characteristics. Comparison of risk scores across (a) WHO grades; (b) different age groups; (c) *IDH1* statuses, (d) 1p/19q codeletion statuses; (e) MGMT promoter methylation statuses.

We further explored the prognostic value of the signature in glioma subgroups with various pathological and molecular features. Stratification analysis was carried out according to WHO grade, *IDH1* status, 1p/19q codeletion status, and O6-methylguanine–deoxyribonucleic acid methyltransferase (MGMT) promoter methylation status, which were significantly related to OS. In all cohorts, high-risk patients had shorter OS than low-risk ones (Figures 5(a)–5(i)). These results indicated that classification based on the immune signature could accurately identify patients with discouraging prognoses, irrespective of pathological and molecular characteristics. In addition, we analyzed differences in risk scores in glioma subgroups. These results showed that patients with age ≥50 years, high grade, wild-type *IDH1*, 1p/19q noncodeletion, and MGMT promoter unmethylated status had higher risk scores than those with age <50 years, low grade, mutated *IDH1*, 1p/19q codeletion, and MGMT promoter methylated status (Figure 6).

### 3.6. Relationship between Risk Score and Infiltrating Immune Cells

**Figure 7:**
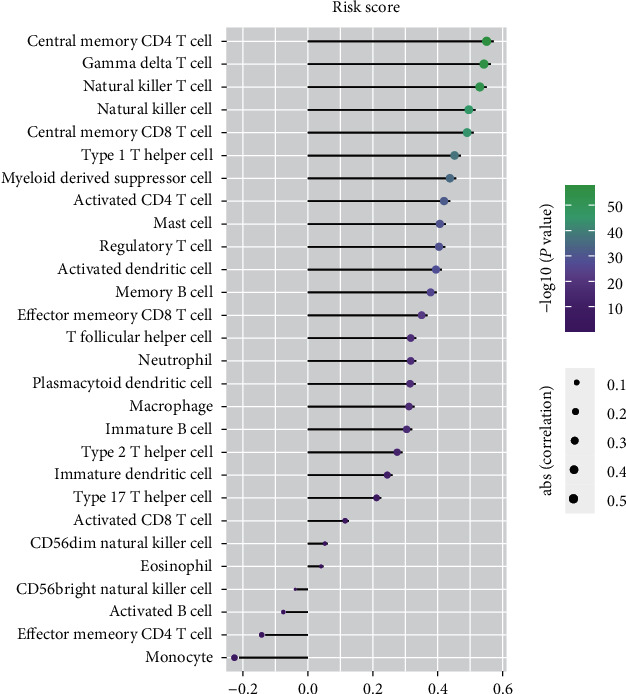
Correlation between the relative abundances of 28 immune cells and signature risk score.

We further analyzed correlations between the risk score of the 15-gene signature and abundances of immune cells. The risk score was positively correlated with abundances of most immune cells, such as central-memory CD4 T cells and *γδ* T cells, and negatively correlated with those of monocytes, effector memory CD4 T cells, activated B cells, and CD56^bright^ NK cells. This further proved that the signature had the potential to reflect the TME characteristics of glioma (Figure 7).

### 3.7. Identification of Biological Function and Signaling Pathways Related to the Prognostic Signature

**Figure 8:**
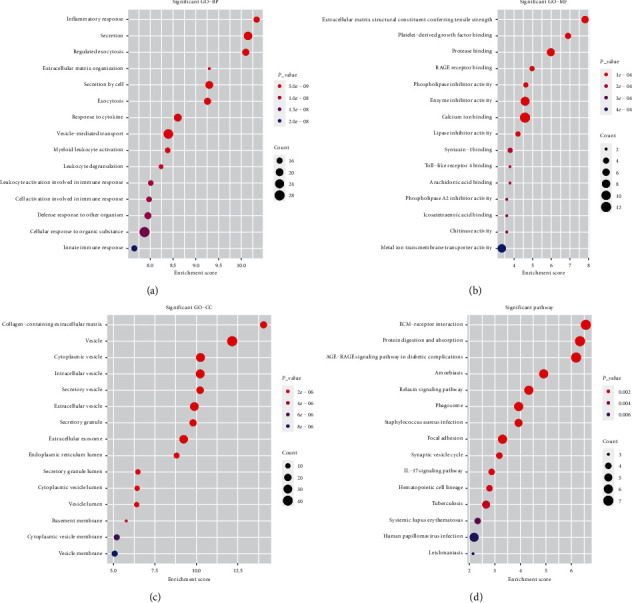
Function and pathway enrichment analyses of the differentially expressed genes. (a) Top 15 enriched terms of biological process. (b) Top 15 enriched terms of molecular function. (c) Top 15 enriched terms of cellular component. (d) Top 15 enriched terms of KEGG pathways.

**Figure 9:**
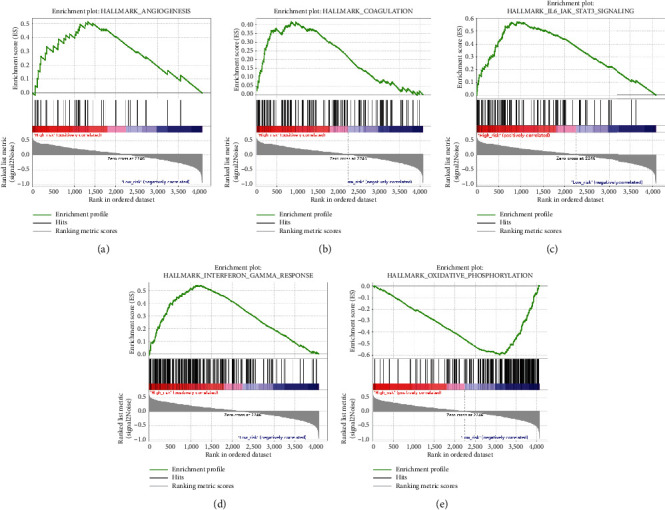
Significant enriched pathways of the low- and high-risk groups by GSEA.

We performed GO and KEGG analyses to investigate the potential BFs and signaling pathways of the hub genes. The results are shown in Figure 8. For BP in GO analysis, the top three enriched terms were inflammatory response, secretion, and regulated exocytosis; these processes were highly correlated with tumor invasiveness. In terms of CC, genes were highly correlated with processes associated with tumor development, including collagen-containing extracellular matrix (ECM), vesicle, and cytoplasmic vesicle. For MF, processes such as ECM structural constituent conferring tensile strength, platelet-derived growth factor (PDGF) binding, and protease binding were significantly enriched by these genes. In KEGG analysis, the targeted genes were highly enriched in essential pathways closely correlated with cancer progression, such as ECM receptor interaction, protein digestion and absorption, and phagosome. Moreover, we also used GSEA data to identify differences in signaling pathways between the low-risk and high-risk groups (Figure 9). We found that the high-risk group was enriched in interleukin-6 (*IL-6*)/Janus kinase (*JAK*)/signal transducer and activator of transcription (*STAT3*) signaling, interferon gamma (IFN-*γ*) response, angiogenesis, and coagulation, which were mainly concentrated in immune-associated pathways. In contrast, the low-risk group was enriched in oxidative phosphorylation, which was correlated with aerobic metabolism.

### 3.8. Validation of the Signature in an External Independent Cohort

**Figure 10:**
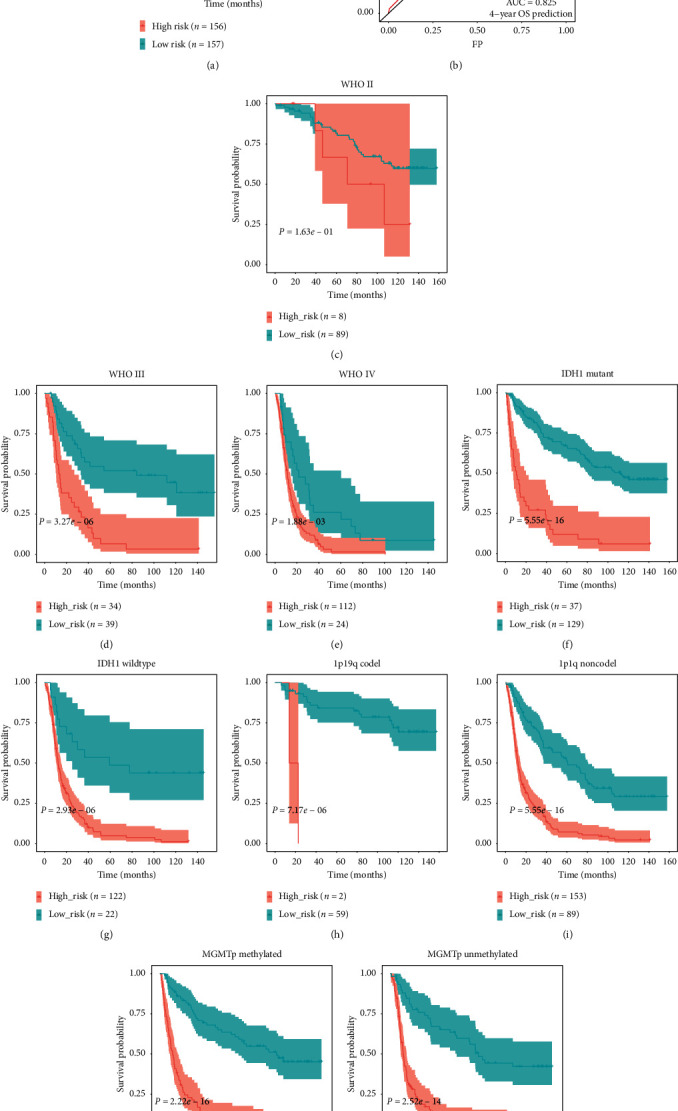
(a) KM survival analysis of the survival difference between the low- and high-risk groups in the external cohort. (b) ROC curve shows the prediction efficiency of the signature in the external cohort. (c–k) Confirmation of the signature via patient stratification based on WHO grade, IDH1 status, 1p/19q codeletion status, and MGMT promoter methylation status in the external cohort.

To validate the robustness of the signature, we calculated patients' risk scores in the external-validation set (CGGA mRNAseq_325). Then, we divided these patients into low- and high-risk groups according to median risk score. KM curve analysis revealed that our signature could significantly distinguish prognosis between the two groups and that the prognoses of patients with high-risk scores were worse than those of patients with low-risk scores (Figure 10(a)). Via ROC analysis, we found that the 4-year AUC was 0.825 in the external cohort, which indicated a good predictive ability (Figure 10(b)). Furthermore, the survival probability of the high- and low-risk groups was significantly different in glioma subgroups stratified by WHO grade (grade III and grade IV), *IDH1* status, 1p/19q codeletion status, and MGMT promoter methylation status (Figures 10(d)–10(k)). However, there was no significant difference in WHO grade II, and the failed statistical analysis was potentially due to limited sample size (Figure 10(c)).

## 4. Discussion

Numerous studies over the past decade have shown that the TME is a critical regulator of tumor progression and therapeutic effectiveness in glioma [32]. Infiltrative inflammatory cells; tissue-resident cells such as astrocytes, microglia, and neurons; and brain vasculature constitute the glioma microenvironment [33]. We investigated the immunogenomic landscape of glioma based on data from the CGGA database and then developed an immune-relevant prognostic gene signature for glioma patients.

As we know, the infiltration of immune cells into the TME reflects immune status and can predict prognosis in cancer [34]. In this study, we evaluated the relative abundance of 28 infiltrating immune cells in the CGGA glioma cohort. There were higher proportions of monocytes, central-memory CD4 T cells, plasmacytoid dendritic cells, CD56^dim^ NK cells, and immature dendritic cells and lower proportions of eosinophils, neutrophils, and activated B cells. Then, we split patients into two subtypes (high_infiltration and low_infiltration) with obviously different infiltration levels of immune cells. It is reported that patients with high immune infiltration tend to have better outcomes than patients with low immune infiltration in bladder, breast, and gastric cancers [35–37]. However, glioma patients in our high-infiltration group showed worse OS than patients in the low-infiltration group. This might have been because immune cells in the glioma microenvironment mainly suppressed immune-mediated destruction of tumor cells [38]. We can also conclude that the grouping method based on the immune landscape was effective in reflecting the degree of immune infiltration and in predicting glioma prognosis. Our prognostic signature was positively correlated with quantity of immunosuppressive cells (such as myeloid-derived suppressor cells, Tregs, and NK T cells) as well as that of immune-stimulatory cells (such as central-memory CD4 T cells, *γδ* T cells, and NK cells) [39, 40]. This result indicated that the regulatory system of the glioma microenvironment was special and complicated, which could also explain the failure of immunotherapy for glioma [41].

To further demonstrate the relationship between the immune landscape of the TME and the prognoses of glioma patients, we constructed an immune-relevant signature by analyzing the CGGA glioma database in detail. A significant difference between the low- and high-risk groups was observed when we applied this 15-gene signature in the training set using ROC and KM curve analyses. The predictive ability of the signature was also validated in the testing set and the external cohort, showing its effectiveness and breadth in predicting prognosis in glioma.

Our results revealed that adenosine triphosphate (ATP)-binding cassette subfamily C member 3 (*ABCC3*), collagen type IV alpha 1 chain (*COL4A1*), podoplanin (*PDPN*), annexin A1 (*ANXA1*), *COL4A2*, insulin-like growth factor binding protein 2 (*IGFBP2*), and *SERPINA3* were positively associated with risk score, while CXXC-type zinc finger protein 11 (*CXXC11*), junctophilin 3 (*JPH3*), secretogranin III (*SCG3*), secreted protein acidic and rich in cysteine (SPARC)-related modular calcium-binding protein 1 (*SMOC1*), Cluster of Differentiation 14 (*CD14*), *COL1A1*, S100 calcium-binding protein A4 (*S100A4*), and transforming growth factor beta 1 (*TGF-β1*) were negatively associated with risk score. Some of these genes have been reported to play essential roles in regulating tumor immune response. For instance, high expression of *PDPN* not only predicts poor survival outcomes but also shows correlations with immune markers of TAMs and T cell exhaustion in gastric cancer [42]. *IGFBP2* induced immunosuppression by decreasing CD8^+^ T and CD19^+^ B cells and increasing CD163^+^ M2 TAMs; blocking this gene suppressed tumor growth and improved survival in a glioblastoma mouse model [43]. *TGF-β* includes three subtypes; Christian *et al.* found that *TGF-β1* and *TGF-β2* mRNAs were strongly expressed in glioblastoma, while *TGF-β3* mRNA was increased particularly in astrocytomas and anaplastic astrocytomas [44]. These subtypes of *TGF-β* play important roles in cancer development processes, including cell invasion, immune suppression, and microenvironment modification [45]. Targeted inhibition of *ANXA1* can reduce the function of Tregs and shrink breast tumors [46]. *COL4A2* expression is positively correlated with the presence of macrophage and dendritic cell infiltration in cervical-cell cancer, while *COL1A1* is positively correlated with tumor infiltration levels of macrophages and CD4^+^ T cells in bladder cancer [47, 48].

LncRNAs have also been reported to play critical roles in the immune response to cancer [49, 50]. We identified 62 differentially expressed lncRNAs correlated with immune infiltration by comparing lncRNA expression levels in the two different immune infiltration groups. Some of these lncRNAs are reported to take part in regulating tumor immune response. *RP5-1119A7.17* is negatively associated with histone 1 (*HIST1*)/histone H2B type 1-K (*H2BK*), which are involved in immune response and cell growth in low-grade glioma [51]. The immunosuppression of CD39^+^ Tregs relies on a distinct transcriptional program with a low level of neurofilament light (*NEFL*) [52]. *NEFL* is also considered an immune gene associated with prognosis in triple-negative breast cancer [53].

Our GSEA results demonstrated that patients in the high-risk group were significantly enriched in immune-associated pathways such as *IL6*/*JAK*/*STAT3* signaling, IFN-*γ* response, angiogenesis, and coagulation. In contrast, patients of the low-risk group were significantly enriched in oxidative phosphorylation. *IL6*/*JAK*/*STAT3* signaling promotes tumor proliferation, invasiveness, and metastasis and suppresses antitumor immune response in the TME [54]. IFN-*γ* response has a controversial role in antitumor immunity. On one hand, IFN-*γ* might suppress the immune response and further contribute to tumor growth and metastasis; on the other hand, it might stimulate host immune response and increase the efficacy of immunotherapy by activating macrophages, dendritic cells, and T lymphocytes in the TME [55]. Previous studies have shown that extravascular coagulation and angiogenesis promote tumor immune evasion and that pharmacological targeting of the coagulation signaling pathway with factor Xa (FXa) could enhance tumor antigen presentation and cytotoxic tumor killing [56–58]. Oxidative phosphorylation is associated with the energy supply of tumor cells. When it is activated, the production of lactic acid is inhibited, which further alkalifies the outside environment and hinders the spread of tumors [59].

Although the immune-related signature was stable in our study, our research had its limitations. First, we excluded patients whose OS was not available, meaning that lost-to-follow-up bias cannot be eliminated. Second, some potential prognostic factors were missing in our signature, such as age, genetic alterations, WHO grade, and treatments. Third, further basic experiments are required to validate the functions of the genes in the signature.

## 5. Conclusion

This investigation comprehensively analyzed RNAseq data from the CGGA database and established an immune-related gene signature with the ability to predict survival outcomes in glioma. We hope that the results of our study can help identify critical genes and pathways associated with glioma and provide potential immune targets for treatment in the future.

## Figures and Tables

**Table 1:** Names of differentially expressed lncRNAs.

Regulation	Differentially expressed lncRNAs
Up high_infiltration vs. low_infiltration	*H19, MIR4435-1HG, AC096579.7, LINC00152, NEAT1, RP11-290F20.3, RP11-1143G9.4, AC015936.3, RP11-161H23.5, RP11-806H10.4, PCED1B-AS1, HOTAIRM1, MIR142, CRNDE, AC011558.5, RP11-344B5.2*
Down high_infiltration vs. low_infiltration	*RP11-161M6.2, AC062021.1, SYN2, LINC00599, RP5-1119A7.17, RP11-231C18.1, RP11-284F21.7, LINC00844, NEFL, RP11-58B17.2, LINC00925, RP11-143K11.1, PWAR6, AC005944.2, RP11-227B21.2, LINC00966, RP3-525N10.2, LINC00689, RP1-293L6.1, MIR7-3HG, MIR219-2, RP11-513I15.6, CTD-2339F6.1, RP11-82C23.2, RP11-401P*9*.4, DGCR5, RP11-355I22.7, RP11-713C5.1, AC004540.4, RP11-1C8.7, RP11-1263C18.1, LINC00461, RP11-571M6.8, RP11-357K6.1, LINC00237, DGCR9, SLC26A4-AS1, RP3-395M20.12, RP11-158M2.3, RP11-1055B8.3, RP11-490M8.1, RP11-127B20.2, RP1-257A7.5, RP5-1177M21.1, AC013402.2, MAPT-AS1*

**Table 2:** Names of differentially expressed mRNAs.

Regulation	Differentially expressed mRNAs
Up high_infiltration vs. low_infiltration	*CHI3L1, CHI3L2, SPOCD1, LTF, SERPINA3, CD163, NNMT, TIMP1, S100A9, COL1A1, IFI30, S100A8, SOCS3, COL3A1, MMP9, TYMP, IGFBP2, C1R, EMP3, PLA2G2A, CP,SPP1, ABCC3, ANXA2, TNFRSF12A, MS4A6A, CD14, FCGBP, S100A4, COL6A2, SLC11A1, ANXA1, TREM1, SERPINE1, SAA1, GPNMB, COL4A1, PDPN, FCGR3A, COL1A2, CCL2, COL4A2, HLA-DRB1, FCGR2B, HLA-DRA, SLC16A3, CD44, GBP2, SERPINA1, TGF-β1*
Down high_infiltration vs. low_infiltration	*NSG2, GRIN1, CPLX2, CHGA, SNAP25, INA, ACTL6B, DLL3, SNCB, SHD, CPLX1, JPH3, CXXC11, CHGB, SMOC1, ATP1A3, KCNIP2, TTC9B, CCK, SCG3, FAM163B, SLC17A7, GDAP1L1, SEZ6L*

**Table 3:** List of genes significantly associated with survival in glioma.

ABCC3, ACTL6B, ANXA1, ANXA2, ATP1A3, C1R, CCK, CCL2, CD14, CD163, CD44, CHGA, CHGB, CHI3L1, CHI3L2, COL1A1, COL1A2, COL3A1, COL4A1, COL4A2, COL6A2, CP, CPLX1, CPLX2, CXXC11, DLL3, EMP3, FAM163 B, FCGBP, FCGR2B, FCGR3A, GBP2, GDAP1L1, GPNMB, GRIN1, HLA-DRA, HLA-DRB1, IFI30, IGFBP2, INA, JPH3, KCNIP2, LTF, MMP9, MS4A6A, NNMT, NSG2, PDPN, PLA2G2A, S100A4, S100A8, S100A9, SCG3, SERPINA1, SERPINA3, SERPINE1, SEZ6L, SHD, SLC11A1, SLC16A3, SMOC1, SNAP25, SNCB, SOCS3, SPOCD1, SPP1, TGF-*β*1, TNFRSF12A, TREM1, TTC9B

**Table 4:** List of fifteen immune-related genes in the prognostic signature.

Gene	Full name	LASSO coefficient
*ABCC3*	Adenosine triphosphate (ATP)-binding cassette subfamily C member 3	0.112657345667762311
*ANXA1*	Annexin A1	0.000508196143922419
*CD14*	Cluster of Differentiation 14 molecule	−0.000638057774923529
*COL1A1*	Collagen type I alpha 1 chain	−0.000274296496641211
*COL4A1*	Collagen type IV alpha 1 chain	0.00253404472560216
*COL4A2*	Collagen type IV alpha 2 chain	6.78971049040963e-05
*CXXC11 (FBXL19)*	F-box and leucine rich repeat protein 19	−0.006215126732245
*IGFBP2*	Insulin-like growth factor binding protein 2	0.000278261058057258
*JPH3*	Junctophilin 3	−0.00464972005220333
*PDPN*	Podoplanin	0.000199537400824582
*S100A4*	S100 calcium-binding protein A4	−0.000619019747732756
*SCG3*	Secretogranin III	−0.00220021211462255
*SERPINA3*	Serpin family A member 3	7.56251372522375e-05
*SMOC1*	SPARC related modular calcium-binding 1	−0.000729769056947777
*TGF-β1*	Transforming growth factor beta 1	−0.000137829618360017

## Data Availability

The transcriptomic and clinical data used to support this study are deposited in CGGA (http://www.cgga.org.cn/).

## Supplementary Materials

Supplementary Materials Figure S1: the KM curves for overall survival of candidate genes in glioma.
